# Effects of Drought and Host on the Growth of *Santalum album* Seedlings in Pot Culture

**DOI:** 10.3390/ijms231911241

**Published:** 2022-09-24

**Authors:** Qilei Zhang, Xiaojin Liu, Daping Xu, Zhou Hong, Ningnan Zhang, Zhiyi Cui

**Affiliations:** Research Institute of Tropical Forestry, Chinese Academy of Forestry, Guangzhou 510520, China

**Keywords:** *Santalum album*, drought, photosynthesis, semi-parasitism

## Abstract

*Santalum album* is a semi parasitic plant and its growth is often restricted due to a lack of a host or water during plantation establishment. In this study, the effects of water and the host on the growth of *S. album* seedlings were studied in pot culture. The results showed that the net photosynthetic rate and height of *S. album* seedlings decreased significantly under drought stress. Compared with the seedlings of *S. album* grown without a host, the host could significantly increase the growth of *S. album* seedlings. The contents of soluble sugar and proline in *S. album* leaves increased significantly under drought stress. Drought stress resulted in a significant accumulation of malondialdehyde, increments of antioxidant enzymes activity, and non-enzymatic antioxidant substances. Antioxidant capacity was stronger and malondialdehyde content was lower in the seedling leaves of *S. album* with a host than in the seedlings without a host. RNA-seq was used to analyze the transcription expression profiles of *S. album* leaves and the results were consistent with the physiological data. These results indicate that the host can promote the seedling growth of *S. album* and it can increase the antioxidant capacity and osmotic adjustment substance content of the seedlings of *S. album*, alleviating the damage caused by drought.

## 1. Introduction

*Santalum album* L., belonging to the family Santalaceae, is a small evergreen tree with semiparasitic roots. At present, it is mainly distributed or planted in humid and hot areas, such as India, Indonesia, Sri Lanka, Malaysia, Southern China, Northern Australia, the Philippines, and some islands in the Pacific Ocean [1]. *S. album* is an economically important tree species because its heartwood is used for carving, incense, perfume, and medicine [2,3,4]. *S. album* has semiparasitic characteristics and its root system must be parasitic on the appropriate host plant root. It needs to obtain necessary water, inorganic salts, and other nutrients through the haustorium connected with the xylem of the host plant root [5].

It has been reported that the germination of *S. album* seeds and the early growth of seedlings do not require the participation of host plants. However, in the subsequent seedling growth process, its root system must parasitize the roots of appropriate host plants; otherwise, *S. album* will not grow in a healthy manner and ultimately dies. Planting hosts with *S. album* seedlings could significantly increase the total lengths, surface areas, and dry weights of *S. album* seedling roots and the total N, total P, and total K contents in whole plants [6]. Radomiljac et al. [7] found that the longer *S. album* seedlings and hosts grow together in a nursery before planting in the field, the larger the height, stem diameter, total plant dry weight, and shoot and root dry weight were after planting in the field. Different hosts have a great influence on the growth of *S. album* seedlings [8]. Host species were found to influence the connections, haustorial numbers, chlorophyll contents, and sizes of *S. album* seedlings, and *S. album* trees grown with suitable hosts exhibited better growth. The study found that N and parasitism had significant interactive effects on total biomass amounts [9]. *S. album* seedlings grown with an N-fixing host exhibited higher net photosynthetic rates and biomass [8].

Plants are subjected to different environmental stresses during their growth, and drought is one of the most common abiotic stresses. Water is a necessary substance for plant growth and development. Lack of water will have an adverse impact on plant growth and development. Drought stress can affect many metabolic and physiological processes of plants, and thus can cause disorders of the plant physiological metabolism system, lead to accumulations of peroxides and reactive oxygen species (ROS) in plant bodies, cause changes in cell membrane peroxidation, protein oxidation, and membrane permeability, and finally affect the normal development of plants. Water is the medium for enzymatic reactions in plant cells. Drought leads to decreases in enzyme activity and metabolic activity in plants and then inhibits plant growth and development [10]. Excessive water loss from cells will lead to the loss of membrane selective permeability and the destruction of the compartmentalization of the inner membrane system, which results in disorders of the normal metabolism in plants. Under drought stress, chlorophyll, nucleic acids, proteins, and other substances are destroyed, enzyme activities decrease, biofilm structures are damaged, stomatal conductance decreases, stomata close, and plant photosynthesis decreases [11,12,13]. Drought stress leads to the obstruction of photosynthesis and causes excess light energy to be obtained by leaves [14]. Moreover, excess amounts of light will form ROS in cells, and large amounts of ROS will destroy cell membranes, cause disorders of the plant cell metabolism, and accelerate plant damage [15,16].

With the rising price of *S. album* heartwood, wild *S. album* resources have been overutilized. At present, *S. album* plantations have been established in large numbers [17,18]. However, after the large-scale planting of *S. album* plantations, seedlings grow slowly or even cannot survive due to a lack of hosts or water. At present, there are few studies on the effects of the host and water on the growth of *S. album* seedlings. Therefore, this study used *S. album* seedlings as experimental materials to study the effects of different soil moisture contents and growth with the host (*Alternanthera bettzickiana*) or not on the growth and development of *S. album* seedlings.

## 2. Results

### 2.1. Increases in Plant Height and Proline, Soluble Sugar, and Leaf Relative Water Contents

**Figure 1:**
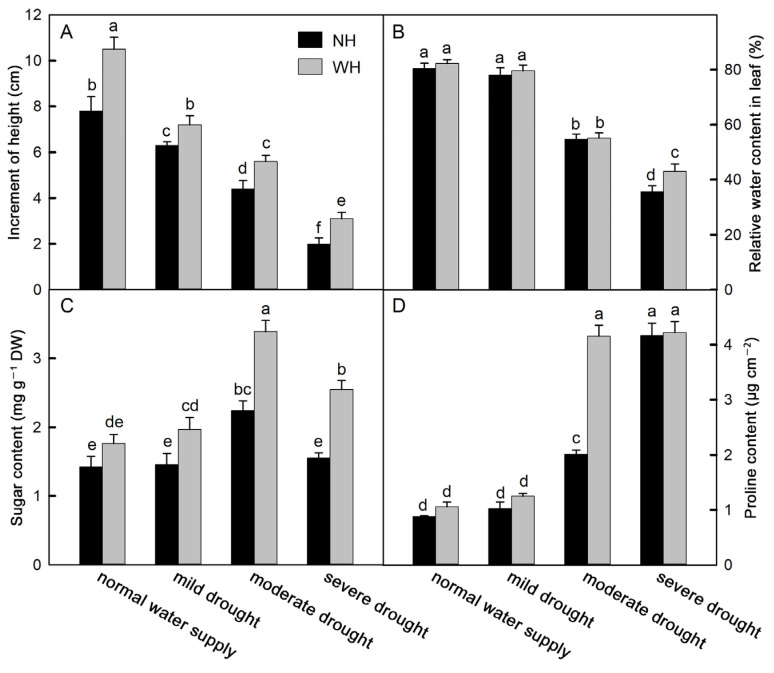
Height, content of proline, and soluble sugar and leaf relative water. Effects of water and host on height change (**A**) (means ± SE, *n* = 10), leaf relative water content (**B**), soluble sugar (**C**), and proline (**D**) content of *S. album* seedling leaves (means ± SE, *n* = 5). NH, grown without host; WH, grown with host. Different letters above bars indicate statistical significance (*p* < 0.05).

Drought stress significantly affected the height growth of *S. album* seedlings. With increased drought stress, seedling heights decreased significantly. Seedlings grown with the host (WH) can significantly increase the height growth of *S. album* seedlings. In the normal watering supply group and under different drought stresses, the increases in *S. album* seedling heights grown with the host were significantly greater than those grown without a host (NH) (Figure 1A). The relative water contents of leaves decreased gradually under drought stress and did not decrease significantly under mild drought stress but decreased significantly under moderate and severe drought conditions. Except for with severe drought, there were no significant differences in leaf relative water contents between plants grown with the host and those grown without a host (Figure 1B). Proline and soluble sugars, as osmotic regulators, can regulate the osmotic pressure in plant cells, and their contents increase under drought stress. With increased drought stress, the proline and soluble sugar contents increased gradually, and the soluble sugar contents decreased under severe drought stress. Growing with a host significantly increased the soluble sugar contents in *S. album* seedling leaves under drought stress (Figure 1C,D).

### 2.2. Photosynthetic Capacity

**Figure 2:**
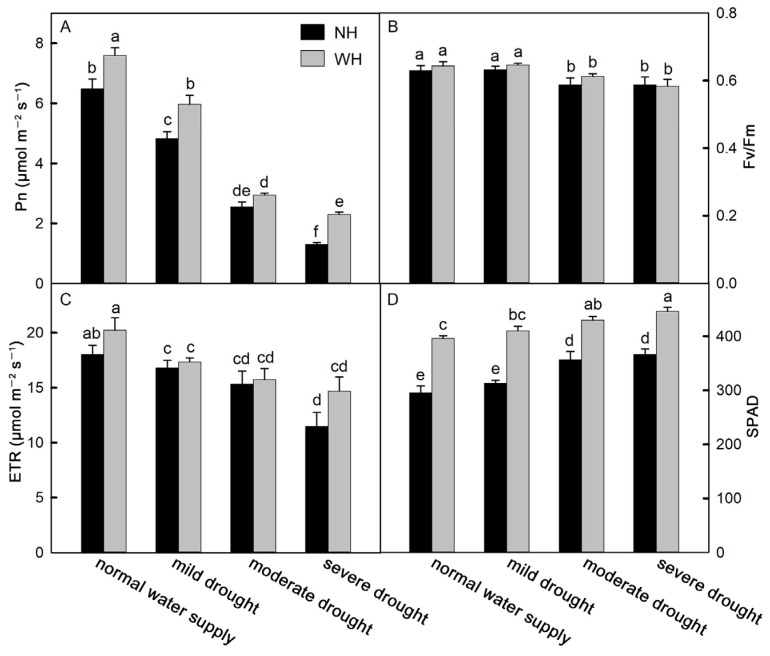
Net photosynthetic rate, maximum photochemical efficiency, electron current transfer rate, and SPAD. Effects of water and host on net photosynthetic rate (Pn, (**A**)), maximum photochemical efficiency (Fv/Fm, (**B**)), electron current transfer rate (ETR, (**C**)), and SPAD (**D**) of *S. album* seedling leaves (means ± SE, *n* = 10). NH, grown without host; WH, grown with host. Different letters above bars indicate statistical significance (*p* < 0.05).

An efficient photosynthetic rate is also conducive to plant growth and development. Compared with *S. album* grown without a host, *S. album* leaves grown with a host showed significantly increased net photosynthetic rates (Pn). With increases in drought stress, the Pn values of *S. album* leaves gradually decreased (Figure 2A). Under mild drought stress, the maximum photochemical efficiency (Fv/Fm) values of *S. album* leaves showed no significant changes between plants grown with the host and those grown without a host and decreased significantly under moderate and severe drought stress, but there were no significant differences between *S. album* under moderate and severe drought stress. There were no significant differences between *S. album* grown with the host and those grown without a host (Figure 2B). Under drought stress, the electron current transfer rate (ETR) of *S. album* leaves decreased significantly, the SPAD of *S. album* leaves increased significantly, and growth with the host significantly increased the SPAD values (Figure 2C,D).

### 2.3. Light Energy Distribution

**Figure 3:**
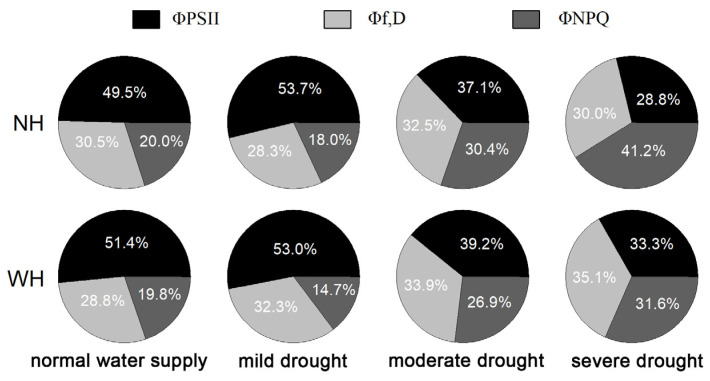
Light energy distribution in leaves of *S. album*. Effects of water and host on light energy distribution of *S. album* seedling leaves (*n* = 10). NH, grown without host; WH, grown with host.

Light energy absorbed by plant leaves is mainly used for photochemical reactions (ΦPSII), nonphotochemical dissipation (Φf,D) and heat dissipation (ΦNPQ). Compared with normal water supply conditions, mild drought stress increased the distribution of light energy in ΦPSII. The proportion of ΦPSII decreased gradually, except under mild drought, and growth with the host increased the proportion of ΦPSII. Under drought stress, Φf,D increased gradually in the leaves of *S. album* grown with the host, but there were no significant changes in these values without a host, and they were higher in leaves of *S. album* grown with the host. Under drought stress, the ΦNPQ values in *S. album* leaves increased gradually, except for LS, and under different drought stress intensities, those grown with hosts were lower than those grown without hosts (Figure 3).

### 2.4. Antioxidant Capacity

**Figure 4:**
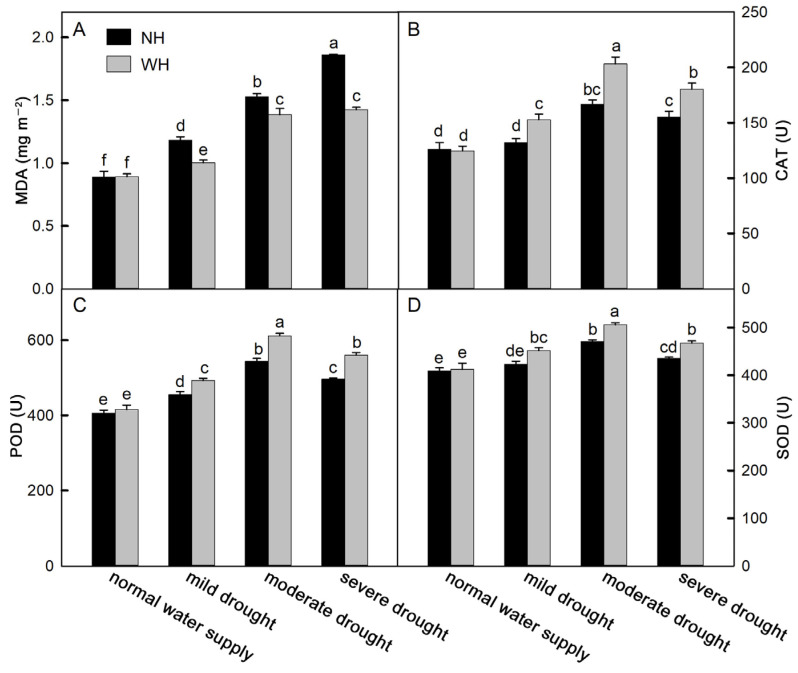
Malondialdehyde (MDA) content and antioxidant enzyme activity. Effects of water and host on MDA content (**A**), activities of catalase (CAT, (**B**)), peroxidase (POD, (**C**)), and superoxide dismutase (SOD, (**D**)) of *S. album* seedling leaves (means ± SE, *n* = 5). NH, grown without host; WH, grown with host. Different letters above bars indicate statistical significance (*p* < 0.05).

**Figure 5:**
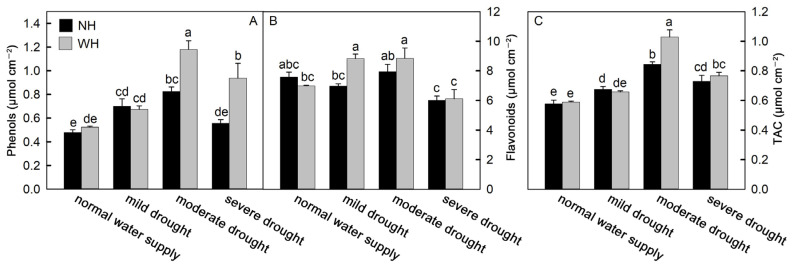
Difference of phenolic and flavonoid contents and total antioxidant capacity. Effects of water and host on content of phenols (**A**) and flavonoids (**B**), and total antioxidant capacity (TAC, (**C**)) of *S. album* seedling leaves (means ± SE, *n* = 5). NH, grown without host; WH, grown with host. Different letters above bars indicate statistical significance (*p* < 0.05).

Drought stress is often accompanied by oxidative stress. As a product of membrane lipid peroxidation, malondialdehyde (MDA) can reflect the degree of oxidative damage to the cell membrane. With an increase in drought stress intensity, the MDA contents in *S. album* leaves increased significantly. Among these, the MDA contents in *S. album* leaves grown without a host increased faster and were higher, which were significantly higher than for *S. album* leaves grown with a host (Figure 4A). To reduce oxidative stress, plant cells can synthesize and accumulate antioxidant substances to remove reactive oxygen species. Antioxidant enzymes are one category of these substances. The results of this experiment show that under drought stress, the activities of catalase (CAT), peroxidase (POD), and superoxide dismutase (SOD) in *S. album* leaves gradually increased, while they decreased under severe drought stress. The antioxidant enzyme activities in the leaves of *S. album* grown with hosts were stronger than those grown without hosts (Figure 4B–D). Flavonoids and phenols, as nonenzymatic antioxidants in plants, can scavenge reactive oxygen species and reduce oxidative stress in plants. In the drought process, the change trends of flavonoids and phenols were consistent with the enzyme activities. The total antioxidant capacity of *S. album* leaves also first increased and then decreased with increasing drought stress intensity (Figure 5).

### 2.5. Number of Differentially Expressed Genes

**Figure 6:**
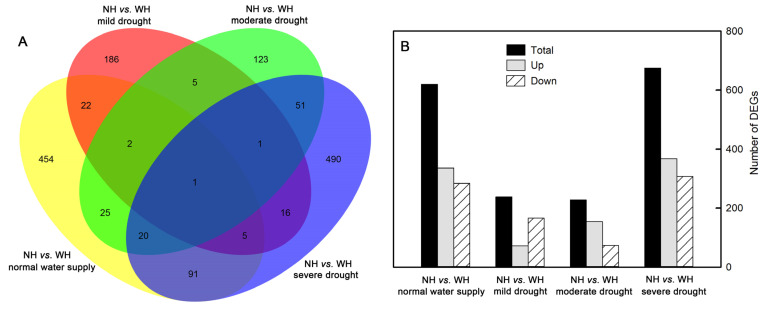
Number of differentially expressed genes (DEGs). Venn diagram showing the number of DEGs (**A**) and uumber of DEGs up-regulated and down-regulated (**B**) between grown without host (NH) and grown with host (WH) under normal water supply, mild drought stress, moderate drought stress, and severe drought stress conditions.

There were 620 differentially expressed genes (DEGs) in *S. album* leaves between the grown without host (NH) and grown with host (WH) under normal water supply conditions, including 336 upregulated genes and 284 downregulated genes. There were 238 DEGs in *S. album* leaves between NH and WH under mild drought stress, including 72 upregulated genes and 166 downregulated genes. There were 228 DEGs in *S. album* leaves between NH and WH under moderate drought stress, including 154 upregulated genes and 74 downregulated genes. There were 675 DEGs in *S. album* leaves between NH and WH under severe drought stress, including 368 upregulated genes and 307 downregulated genes (Figure 6).

### 2.6. Gene Expressions between Plants Grown without Host and Those Grown with Host under Different Moisture Conditions

**Figure 7:**
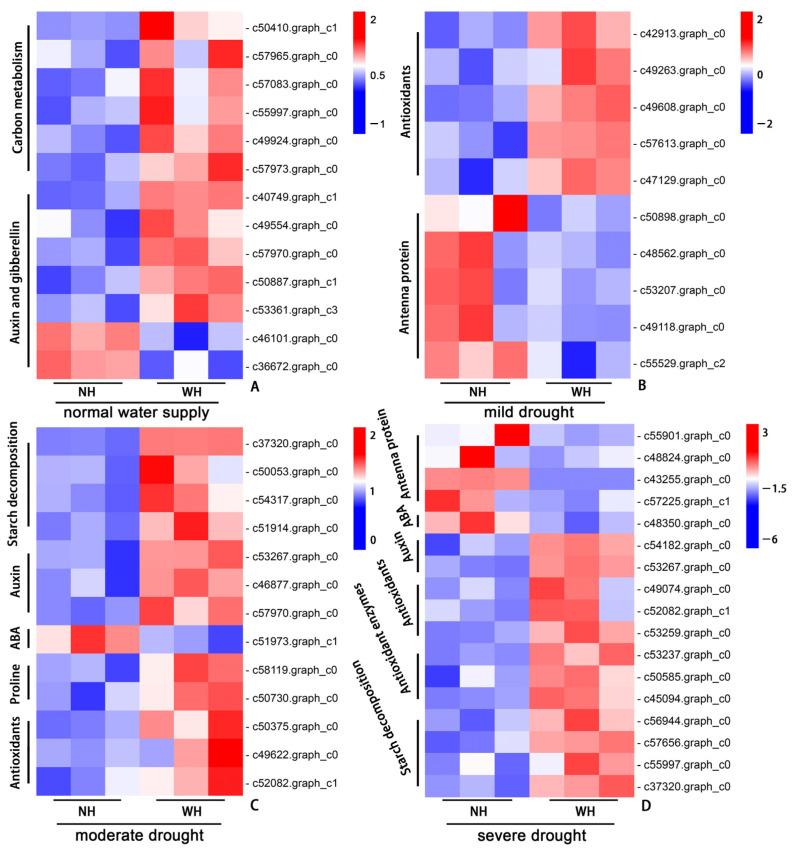
Heatmap of gene expression. Gene expression between grown without host (NH) and grown with host (WH) under normal water supply (**A**), mild drought stress (**B**), moderate drought stress (**C**), and severe drought stress (**D**) conditions.

Combined with the above data analysis and physiological results, we conducted a cluster heatmap analysis on the related pathway genes. Under normal water supply conditions, the production of DEGs was mainly affected by the host. The expressions of pathway genes related to carbon metabolism were upregulated in the leaves of *S. album* grown with hosts. The positive regulation-related genes in the auxin and gibberellin signal transduction pathways were upregulated in the leaves of *S. album* grown with hosts, and the negative regulation-related genes were downregulated (Figure 7A). Under mild drought stress, the DEGs were mainly concentrated in the synthesis of nonenzymatic antioxidant substances and synthesis pathways of photosystem antenna proteins. The genes related to the synthesis of antioxidant substances were upregulated in leaves of *S. album* grown with the host, while the genes related to the synthesis of photosystem antenna proteins were upregulated in the leaves of *S. album* grown without a host (Figure 7B). Under moderate drought stress, the DEGs in *S. album* leaves were mainly concentrated in the starch decomposition, auxin and abscisic acid signal transduction, proline and nonenzymatic antioxidant synthesis pathways. The negative regulatory genes in the abscisic acid signal transduction pathway were downregulated in the leaves of *S. album* grown with hosts, and other pathway genes were upregulated (Figure 7C). Under severe drought stress, the DEGs were mainly concentrated in the starch decomposition, auxin and abscisic acid signal transduction, antenna protein synthesis, antioxidant enzymes, and nonenzymatic antioxidant substance synthesis pathways. The negative regulatory genes in the abscisic acid signal transduction and photosystem antenna protein synthesis pathways were downregulated in leaves of *S. album* grown with hosts, and other pathway genes were upregulated (Figure 7D).

## 3. Discussion

Drought stress reduced the photosynthetic capacity and growth of *S. album* seedlings, and seedlings with hosts were able to alleviate the effects of drought stress. As an important environmental factor, water shortages will directly affect the growth and development of plants [19,20]. The results showed that the heights of *S. album* decreased gradually with increasing levels of drought stress. Compared with seedlings grown without a host, the heights of *S. album* grown with hosts were higher. As semiparasitic plants, hosts can provide nutrients and water for *S. album* [7]. Studies have shown that the growth rate of *S. album* can be increased by accompanying seedlings with hosts, and suitable hosts are more conducive to *S. album* growth than unsuitable hosts [21]. Drought affects plant growth because it reduces the photosynthetic rates of plant photosynthetic organs [22]. Severe drought stress will cause disorders of the plant physiological metabolism system [23]. Figure 2 showed that the more serious the drought stress was, the lower the net photosynthetic rate was.

Fv/Fm is one of the important parameters related to chlorophyll fluorescence in plant leaves. It decreases significantly under stress conditions (e.g., high temperature, low temperature, or drought) [24,25]. The results showed that the Fv/Fm values decreased under drought stress, and Fv/Fm decreased significantly under moderate and severe drought stress. The SPAD values of plant leaves were significantly positively correlated with the chlorophyll and N contents [9]. In this experiment, the SPAD values showed an upward trend with increasing drought stress intensity, which may be because the chlorophyll concentrations increased with decreasing leaf water content (Figure 2D). The SPAD values in the leaves of *S. album* grown with hosts were significantly higher than those without a host, which may be because the hosts provided more N elements to *S. album* [9].

Under drought stress, plants accumulate large quantities of ROS, resulting in oxidative stress [26]. High ROS levels have strong toxic effects on macromolecules, such as plant cell membrane systems, proteins, and nucleic acids [27]. MDA is the product of membrane lipid peroxidation, and its contents in *S. album* leaves grown without hosts were significantly higher than those grown with hosts (Figure 4A). The results suggested that growth with the host could maintain the stability of the membrane structure and improve drought tolerance to a certain extent. The activities of POD, SOD, and CAT in *S. album* leaves increased significantly under mild and moderate drought stress (Figure 4B–D). This may be because the increased ROS accumulations under drought stress led to increased antioxidant enzyme activities in plant cells [28]. Under severe drought stress, the antioxidant enzyme activities decreased, which may have resulted from the serious damage to the *S. album* leaves. The antioxidant enzyme activities in *S. album* leaves grown with the host were significantly stronger than those grown without a host. Deepa and Yusuf also confirmed that the host could improve the enzyme activities in *S. album* leaves [6].

Under drought stress, cells can rapidly synthesize and accumulate osmotic protectants, including sugar and proline. These osmotic cells help rebuild the cell osmotic balance by increasing the cell water potential, and thus play a key role in protecting and stabilizing organelles [29]. Drought stress increased the proline and soluble sugar contents in *S. album* leaves, and they were higher in *S. album* leaves grown with hosts (Figure 1C,D). In a study of maize, it was also found that drought stress could significantly increase the proline and soluble sugar contents in maize cells, and maize lines with higher contents could better maintain cell turgor [30,31].

According to the transcriptome data, under normal water supply conditions, the DEGs between plants grown with hosts and those grown without hosts were mainly related to carbon metabolism, auxin, and gibberellin. The gene expression levels of *S. album* grown with hosts were significantly upregulated, and the gene expressions related to auxin negative regulation were downregulated, which may be because the host promoted *S. album* growth [9]. Under mild drought conditions, the DEGs were mainly concentrated in the antioxidant substances and the antenna protein synthesis pathways. Genes related to the synthesis of antioxidant substances were upregulated in *S. album* leaves grown with hosts, which was consistent with the physiological results. The host could promote the synthesis of antioxidant substances, improve antioxidant capacities, and decrease the damage caused by drought stress. Genes related to antenna protein synthesis were downregulated to reduce the absorption of light energy by leaves. Drought stress can significantly affect the carbon reaction process in photosynthesis, and the utilization of light energy decreases significantly [32]. Excess light energy that cannot be effectively utilized will lead to the production of large amounts of ROS. Therefore, the decreased absorption of light energy by antenna proteins under drought stress has a protective effect on plant leaves. Under moderate and severe drought conditions, genes related to starch decomposition and proline synthesis were upregulated in *S. album* leaves grown with hosts. Proline synthesis is conducive to the production of osmotic regulators in cells and improves the resistance of plants to drought stress [33]. The positive regulatory genes in the auxin signal transduction pathway and genes related to the synthesis of antioxidant enzymes and nonenzymatic antioxidant substances pathways were also upregulated. The negative regulatory genes in the ABA signal transduction pathway were downregulated in *S. album* leaves grown with the host, which was conducive to the response of downstream genes in the ABA signal transduction pathway. ABA is considered to be the hormone that is most closely related to the drought stress response in plants and plays a key role in coordinating the drought stress response [23,34]. 

## 4. Materials and Methods

### 4.1. Plant Materials

In the pot experiment, *S. album* seedlings were planted in plastic pots (17.4 cm high, 18.8 cm upper diameter, 13.5 cm lower diameter) containing growth substrate (red soil: sand = 3:1, *v*/*v*). Under mild drought stress (soil relative water content is 55–60%); moderate drought stress (soil relative water content is 40–45%); severe drought stress (soil relative water content is 20–25%); and normal water supply (soil relative water content is 75–80%), the soil water contents were monitored by the weighing method. The relative water content gradients of the four soils all included those grown with hosts (*Alternanthera bettzickiana*) and those grown without hosts. During the experiment, the average temperature in the greenhouse during the day was 23–32 °C, the relative air humidity was 45–85%, and natural sunlight was provided. The relevant indices were determined after 90 days of treatment. 

### 4.2. Leaf Water Contents

The water contents of leaves examined in the experiment consisted of the relative water contents, and the measurement method refers to Zhang et al. [20]. *S. album* leaves were cut and weighed (FW), immersed in water for 24 h to fully absorb water, saturated and weighed (TW), placed in an oven at 105 °C for 30 min, dried at 75 °C to constant weight, and weighed (DW). The calculation formula for the blade relative water content is RWC = (FW − DW)/(TW − DW) × 100%.

### 4.3. Determinations of Net Photosynthetic Rates, Chlorophyll Fluorescence Parameters and Light Energy Distributions

The net photosynthetic rates (Pn) were measured according to Liu et al. [35]. A portable Photosynthesis System LI-6800 (LI-COR, Lincoln, NE, USA) was used to determine the Pn values of leaves. The light intensity of the detection blade chamber was set to 800 μmol m^−2^ s^−1^, the ratio of red to blue light was nine to one, the CO_2_ concentration was 400 μmol m^−2^ s^−1^, the humidity was 65%, and the temperature was 28 °C. The Pn values of *S. album* leaves were recorded after they became relatively stable.

*S. album* leaves were wrapped with tin foil for dark adaptation for 30 min. After dark adaptation, a portable fluorometer (PAM-2500, Heinz Walz, Effeltrich, Germany) was used with the photochemical intensity set to 800 μmol m^−2^ s^−1^ to determine the chlorophyll fluorescence parameters, and the calculation method of Baker [36] was used.

MultispeQ V2.0 was used to determine the light energy distribution of plant leaves. Sunny days were selected for conducting the measurements, PhotosynQ software was installed on a mobile phone, which was connected with the instrument through Bluetooth, a clamp was used to fix plant leaves in a leaf chamber, the mobile phone was clicked, which was followed by a pause before a measurement was obtained and was clicked again to save the data. During the measurement process, the optical quantum sensor was not blocked, and the growth angles of the leaves were not changed [37].

### 4.4. Soluble Sugar and Proline Content

The blades were washed with ultrapure water three times, heated at 105 °C for 20 min, and dried at 70 °C to constant weight. The soluble sugar contents were determined according to the method described by Zhang et al. [20]. The soluble sugar contents in the samples were calculated by using different concentrations of glucose as the standard curve.

Six leaf discs (7 mm in diameter) were placed into a mortar and 10 mL of 80% ethanol was added for grinding. Then, the grinding liquid was poured into 0.01 g of activated carbon and extracted in the dark for 1 h. The proline contents were determined according to the method described by Zhang et al. [20]. The proline concentrations were calculated by using different proline concentrations as the standard curve.

### 4.5. Activities of Catalase, Superoxide Dismutase and Peroxidase

Six leaf discs (7 mm in diameter) from each treatment were collected for CAT, SOD, and POD analyses at the end of the experiment. The CAT, SOD, and POD activities were measured as described by Yang et al. [38].

### 4.6. Determinations of Malondialdehyde, Flavonoids, Total Phenols and Total Antioxidant Capacity

The malondialdehyde (MDA) contents were determined according to the method of Sun et al. [39]. Six leaf discs (7 mm in diameter) were placed into a mortar, 2 mL of 10% trichloroacetic acid was added for grinding, centrifuging at 4000× *g* at 4 °C for 15 min was applied, 1.5 mL of supernatant was aspirated and was mixed with 0.67% 2-thiobarbituric acid of the same volume and then boiled for 20 min. After cooling, the absorbance values were measured at 600, 532, and 450 nm with a UV spectrophotometer UV-2450 (Shimadzu, Japan).

Two leaf discs (7 mm in diameter) were immersed overnight in 1.5 mL of 95% methanol at 4 °C to obtain flavonoids and a total phenol extract. The method of flavonoid determination described by Heimler et al. [40] was slightly modified. A 0.5-mL sample solution was diluted 10 times, 0.2 mL of 5% NaNO_2_, 0.3 mL of prepared 10% AlCl_3_, and 1 mL of 1 M NaOH were added in sequence, and finally, the volume was fixed to 3.5 mL with deionized water. After mixing, the absorbance values were measured at 510 nm. A standard curve was established with catechin, and the total flavonoid contents in the samples were calculated.

The total phenol contents were determined with a Folin phenol reagent [41]. Starting with 0.5 mL of a 20-times diluted sample solution, 1 mL of 10% Folin phenol reagent was first added, and 2 mL of 0.7 M Na_2_CO_3_ was then added. After mixing, the absorbances at 765 nm were determined. Gallic acid was used to establish the standard curve and to calculate the total phenol contents.

The total antioxidant capacities were determined by the scavenging reaction of DPPH radical (1,1-diphenyl-2-trinitrophenylhydrazine) according to the steps described by Saha et al. [42]. A DPPH solution (100 µmol L^−1^) was prepared with 95% methanol. A 0.3-mL sample solution was mixed with 2.7 mL of DPPH solution and allowed to stand at room temperature for 5 min. Then, 95% methanol was used as the blank control to determine the absorbances at 517 nm. The absorbance changes at 517 nm were determined, a standard curve was established, and the ability of the sample to remove DPPH was calculated.

### 4.7. Total RNA Extraction and RNA Sequencing

For the transcriptome analysis, *S. album* leaves were collected under the treatment conditions as described above in three biological replicates. Total RNA was extracted from the leaves of *S. album* seedlings by the TRIzol reagent (Invitrogen, MA, USA). The cDNA was amplified using M-MLV reverse transcriptase (Takara, Tokyo, Japan). An Illumina HiSeq 2000 platform was used to perform paired-end sequencing. RNA sequencing data were available in the Gene Expression Omnibus (GEO) database under accession number SUB11612273. Functional annotation on sequencing results was carried out according to the method described by Zhang et al. [43].

### 4.8. Functional Annotation and Identification of Differentially Expressed Genes

To obtain the direction, function, and pathway annotations of unigenes, the unigenes of two species were searched against the nonredundant protein (Nr) database, Swiss-Prot protein database, and the Kyoto Encyclopedia of Genes and Genomes (KEGG) pathway database using BLASTx with an E-value cut-off of <10^−5^. A gene ontology (GO) annotation of unigenes for describing biological processes, molecular functions, and cellular components was performed using Blast2GO software.

The abundance of each transcript was calculated based on the number of clean reads mapped to the reference unigenes using Bowtie 2 and eXpress software, as well as the FPKM (fragments per kilobase per million reads) method [44]. The DEGseq package was used to generate the ‘baseMean’ values and identify the differentially expressed genes (DEGs). The *p* value ≤ 0.01 and an absolute value of log2FoldChange ≥ 1.5 were used as the threshold for identifying genes that were significantly differentially expressed in paired comparison samples.

### 4.9. Statistical Analysis

Data were subjected to a one-way analysis of variance (ANOVA) with Duncan’s multiple range test to compare means using SPSS Statistics 19.0 (IBM, Armonk, NY, USA), with a significance set at two-tailed *p* < 0.05. All analyses were conducted using SigmaPlot version 12.5 (SYSTAT Software, Richmond, CA, USA).

## References

[1] Ma G.H., Bunn E., Zhang J.F., Wu G.J. (2006). Evidence of dichogamy in *Santalum album* L.. J. Integr. Plant. Biol.

[2] Arasada B.L., Bommareddy A., Zhang X.Y., Bremmon K., Dwivedi C. (2008). Effects of alpha-santalol on proapoptotic caspases and p53 expression in UVB irradiated mouse skin. Anticancer Res..

[3] Bhatia S.P., McGinty D., Letizia C.S., Api A.M. (2008). Fragrance material review on alpha-santalol. Food Chem. Toxicol..

[4] Burdock G.A., Carabin L.G. (2008). Safety assessment of sandalwood oil (*Santalum album* L.). Food Chem. Toxicol..

[5] Yang X., Zhang X., da Silva J.A.T., Liang K., Deng R., Ma G. (2014). Ontogenesis of the collapsed layer during haustorium development in the root hemi-parasite Santalum album Linn. Plant. Biol..

[6] Deepa P., Yusuf A. (2016). Influence of different host associations on glutamine synthetase activity and ammonium transporter in *Santalum album* L.. Physiol. Mol. Biol. Pla..

[7] Radomiljac A.M., McComb J.A., Shea S.R. (1998). Field establishment of *Santalum album* L.-the effect of the time of introduction of a pot host (Alternanthera nana R. Br.). Forest Ecol. Manag..

[8] Balasubramanian A., Prasath C.N.H., Radhakrishnan S., Sivaprakash M. (2021). Host-specific influence on early growth and physiological attributes of sandal (*Santalum album*) grown in farmlands. J. Environ. Biol..

[9] Meng S., Ma H.B., Li Z.S., Yang F.C., Wang S.K., Lu J.K. (2021). Impacts of nitrogen on physiological interactions of the hemiparasitic *Santalum album* and its N-2-fixing host Dalbergia odorifera. Trees-Struct. Funct..

[10] Ramegowda V., Senthil-Kumar M. (2015). The interactive effects of simultaneous biotic and abiotic stresses on plants: Mechanistic understanding from drought and pathogen combination. J. Plant. Physiol..

[11] De Vries F.T., Griffiths R.I., Knight C.G., Nicolitch O., Williams A. (2020). Harnessing rhizosphere microbiomes for drought-resilient crop production. Science.

[12] Soares-Cordeiro A.S., Carmo-Silva A.E., da Silva A., da Silva J., Keys A.J., Arrabaca M.C. (2009). Effects of rapidly imposed water deficit on photosynthetic parameters of three C-4 grasses. Photosynthetica.

[13] Xu X., Peng G.Q., Wu C.C., Korpelainen H., Li C.Y. (2008). Drought inhibits photosynthetic capacity more in females than in males of Populus cathayana. Tree Physiol..

[14] Barber J., Andersson B. (1992). Too Much of a Good Thing-Light Can Be Bad for Photosynthesis. Trends Biochem. Sci..

[15] Murata N., Allakhverdiev S.I., Nishiyama Y. (2012). The mechanism of photoinhibition in vivo: Re-evaluation of the roles of catalase, alpha-tocopherol, non-photochemical quenching, and electron transport. Bba-Bioenergetics.

[16] Zhang T.J., Zheng J., Yu Z.C., Gu X.Q., Tian X.S., Peng C.L., Chow W.S. (2018). Variations in photoprotective potential along gradients of leaf development and plant succession in subtropical forests under contrasting irradiances. Environ. Exp. Bot..

[17] Da Silva J.A.T., Kher M.M., Soner D., Page T., Zhang X.H., Nataraj M., Ma G.H. (2016). Sandalwood: Basic biology, tissue culture, and genetic transformation. Planta.

[18] Rashkow E.D. (2014). Perfumed the axe that laid it low: The endangerment of sandalwood in southern India. Indian Econ. Soc. Hist..

[19] Kaur N., Gupta A.K. (2005). Signal transduction pathways under abiotic stresses in plants. Curr. Sci. India.

[20] Zhang Q.L., Huang J.D., Ke W.Q., Cai M.L., Chen G.X., Peng C.L. (2021). Responses of *Sphagneticola trilobata*, *Sphagneticola calendulacea* and their hybrid to drought stress. Int. J. Mol. Sci..

[21] Ouyang Y., Zhang X.H., Chen Y.L., da Silva J.A.T., Ma G.H. (2016). Growth, photosynthesis and haustorial development of semiparasitic *Santalum album* L. penetrating into roots of three hosts: A comparative study. Trees-Struct. Funct..

[22] Sadak M.S., Abdalla A.M., Abd Elhamid E.M., Ezzo M.I. (2020). Role of melatonin in improving growth, yield quantity and quality of *Moringa oleifera* L. plant under drought stress. Bull. Natl. Res. Cent..

[23] Takahashi F., Suzuki T., Osakabe Y., Betsuyaku S., Kondo Y., Dohmae N., Fukuda H., Yamaguchi-Shinozaki K., Shinozaki K. (2018). A small peptide modulates stomatal control via abscisic acid in long-distance signalling. Nature.

[24] Guadagno C.R., Ewers B.E., Speckman H.N., Aston T.L., Huhn B.J., DeVore S.B., Ladwig J.T., Strawn R.N., Weinig C. (2017). Dead or Alive? Using Membrane Failure and Chlorophyll a Fluorescence to Predict Plant Mortality from Drought. Plant Physiol..

[25] Sun L.Y., Li X.N., Wang Z.S., Sun Z.W., Zhu X.C., Liu S.Q., Song F.B., Liu F.L., Wang Y.J. (2018). Cold Priming Induced Tolerance to Subsequent Low Temperature Stress is Enhanced by Melatonin Application during Recovery in Wheat. Molecules.

[26] Wu H., Fu B., Sun P.P., Xiao C., Liu J.H. (2016). A NA.AC Transcription Factor Represses Putrescine Biosynthesis and Affects Drought Tolerance. Plant Physiol..

[27] Zhang Y.J., Zhou G.S. (2011). Exploring the effects of water on vegetation change and net primary productivity along the IGBP Northeast China Transect. Environ. Earth Sci..

[28] Bashir W., Anwar S., Zhao Q., Hussain I., Xie F. (2019). Interactive effect of drought and cadmium stress on soybean root morphology and gene expression. Ecotox. Environ. Safe..

[29] Khan M.S., Ahmad D., Khan M.A. (2015). Utilization of genes encoding osmoprotectants in transgenic plants for enhanced abiotic stress tolerance. Electron. J. Biotechnol..

[30] Alharby H.F., Fahad S. (2020). Melatonin application enhances biochar efficiency for drought tolerance in maize varieties: Modifications in physio-biochemical machinery. Agron. J..

[31] Yasmin H., Rashid U., Hassan M.N., Nosheen A., Naz R., Ilyas N., Sajjad M., Azmat A., Alyemeni M.N. (2021). Volatile organic compounds produced by Pseudomonas pseudoalcaligenes alleviated drought stress by modulating defense system in maize (*Zea mays* L.). Physiol Plant..

[32] Zhang X.B., Lei L., Lai J.S., Zhao H.M., Song W.B. (2018). Effects of drought stress and water recovery on physiological responses and gene expression in maize seedlings. Bmc Plant. Biol..

[33] Hayat S., Hayat Q., Alyemeni M.N., Wani A.S., Pichtel J., Ahmad A. (2012). Role of proline under changing environments: A review. Plant. Signal. Behav..

[34] Barrero J.M., Rodriguez P.L., Quesada V., Piqueras P., Ponce M.R., Micol J.L. (2006). Both abscisic acid (ABA)-dependent and ABA-independent pathways govern the induction of NCED3, AAO3 and ABA1 in response to salt stress. Plant. Cell Environ..

[35] Liu X.J., Xu D.P., Yang Z.J., Zhang N.N., Pan L.J. (2018). Investigation of Exogenous Benzyladenine on Growth, Biochemical Composition, Photosynthesis and Antioxidant Activity of Indian Sandalwood (*Santalum album* L.) Seedlings. J. Plant. Growth Regul..

[36] Baker N.R. (2008). Chlorophyll fluorescence: A probe of photosynthesis in vivo. Annu. Rev. Plant. Biol..

[37] Kuhlgert S., Austic G., Zegarac R., Osei-Bonsu I., Hoh D., Chilvers M.I., Roth M.G., Bi K., TerAvest D., Weebadde P. (2016). MultispeQ Beta: A tool for large-scale plant phenotyping connected to the open PhotosynQ network. Roy. Soc. Open Sci..

[38] Yang F., Wang Y., Wang J., Deng W.Q., Liao L., Li M. (2011). Different eco-physiological responses between male and female Populus deltoides clones to waterlogging stress. Forest Ecol. Manag..

[39] Sun Z.Y., Chen Y.Q., Schaefer V., Liang H.M., Li W.H., Huang S.Q., Peng C.L. (2015). Responses of the Hybrid between Sphagneticola trilobata and Sphagneticola calendulacea to Low Temperature and Weak Light Characteristic in South China. Sci. Rep..

[40] Heimler D., Vignolini P., Dini M.G., Romani A. (2005). Rapid tests to assess the antioxidant activity of *Phaseolus vulgaris* L. dry beans. J. Agr. Food Chem..

[41] Ainsworth E.A., Gillespie K.M. (2007). Estimation of total phenolic content and other oxidation substrates in plant tissues using Folin-Ciocalteu reagent. Nat. Protoc..

[42] Saha M.R., Hasan S.M.R., Akter R., Hossain M.M., Alam M.S., Alam M.A., Mazumder M.E.H. (2008). In vitro free radical scavenging activity of methanol extract of the leaves of Mimusops elengi Linn. Bangladesh J. Vet. Med..

[43] Zhang T.J., Zheng J., Yu Z.C., Huang X.D., Zhang Q.L., Tian X.S., Peng C.L. (2018). Functional characteristics of phenolic compounds accumulated in young leaves of two subtropical forest tree species of different successional stages. Tree Physiol..

[44] Trapnell C., Williams B.A., Pertea G., Mortazavi A., Kwan G., van Baren M.J., Salzberg S.L., Wold B.J., Pachter L. (2010). Transcript assembly and quantification by RNA-Seq reveals unannotated transcripts and isoform switching during cell differentiation. Nat. Biotechnol..

